# Editorial: Advances and new perspectives in higher education quality

**DOI:** 10.3389/fpsyg.2024.1395604

**Published:** 2024-04-09

**Authors:** Ana B. Bernardo, María Esteban, Ellián Tuero, Joana R. Casanova, Antonio Cervero

**Affiliations:** ^1^Psychology Department, University of Oviedo, Oviedo, Asturias, Spain; ^2^CIEd - Research Centre on Education, Institute of Education, University of Minho, Braga, Portugal

**Keywords:** higher education quality, new teaching methods, student satisfaction, student performance, educational quality indicators

The technological advance that occurred in the last decades of the 20th century generated a transformation in society and its production systems, increasingly demanding more specialized, more qualified, competent, and committed professionals. Consequently, educational systems have been modernizing and democratizing, making tertiary education a possibility within the reach of the majority of young people. To respond to the new demands of the professional market, Higher Education has implemented new degrees, master's degrees, training courses, etc., and launched different quality assurance systems, with the aim of improving the teaching, research, management activity of university centers, etc.

To achieve the objective of improving the quality and efficiency of the university system, it is necessary to carry out research that provides knowledge about the current characteristics of students and university and pre-university institutions, and then, based on them, design more efficient educational processes. In this sense, higher education institutions need validated instruments with high reliability to evaluate their students and better understand their qualities with the aim of providing them with the skills and resources necessary for their academic and professional success. Examples of this type of instruments are the PENCRISAL—dedicated to evaluating critical thinking skills—(Rivas et al.) or the Writing-Specific Cognitive Strategies Questionnaire—dedicated to evaluating preferences regarding writing strategies—(Arias Gundin et al.), whose psychometric properties are explored in this monograph.

However, research has shown how students arrive at university with baggage that -in some way- conditions their learning processes (Wang et al.). Advances in this research field show how the transition period from secondary education to university can be critical for many of them, inclining them to fail or favoring their permanence in the institution. Consequently, the preparation of students in secondary school is of special importance, since this must ensure that they acquire a series of academic skills and psychosocial resources that guarantee their subsequent success. To this end, it is necessary to refer to a prolific line of research that confirms how a large part of students enter the university without being self-regulated students, which hinders their adequate progress in the institution (Saéz-Delgado et al.) On the other hand, and focusing on the university stage, the student's psychological capital has proven to influence the way they approach academic tasks, potentially resulting in greater academic commitment (Palos et al.) and mediating between their personality traits and the development of key competencies (Hu et al.) Similarly, how the student sees his own career can favor his adjustment to the requirements of the university and even prevent him from dropout his university studies (Cobo-Rendón et al.).

Regarding the teaching-learning processes, the university is making real efforts to renew its teaching methodologies and thereby improve the quality of instruction. In this sense, the planification of teaching by competencies is yielding excellent results both in undergraduate studies (Erschens et al.), as well as in postgraduate studies (Ndeezi et al.) and doctorate studies (Mumba et al.). Likewise, other pedagogies such as integrated laboratory practices (Sánchez et al.) or the use of photographs to learn about historical events (Ponsoda-López de Atalaya et al.) generate high student motivation and foster an improvement of educational results.

In relation to the above, it is also necessary to highlight the role of the teaching staff, who after the modernization of the university system plays a fundamental role as facilitators of learning. In relation to this, the research results obtained by Liu et al.—are illustrative—who confirm how adequate feedback favors the motivation, work and creativity of students- or those obtained by Bautista Cruz and Kim, who confirm how a leadership attitude on the part of the teaching staff positively influences the satisfaction of the university student.

The works compiled in this Research Topic are examples of the progress that Higher Education institutions have made toward quality in the development of their functions. Without a doubt, the knowledge generated by these and other research on the topic will contribute to shaping the Higher Education of the future.

## Author contributions

AB: Conceptualization, Funding acquisition, Investigation, Supervision, Validation, Visualization, Writing—review & editing. ME: Conceptualization, Data curation, Formal analysis, Funding acquisition, Investigation, Methodology, Project administration, Resources, Software, Supervision, Validation, Visualization, Writing—original draft, Writing—review & editing. ET: Software, Supervision, Validation, Writing—review & editing. JC: Conceptualization, Data curation, Formal analysis, Investigation, Methodology, Resources, Supervision, Validation, Visualization, Writing—original draft, Writing—review & editing. AC: Conceptualization, Formal analysis, Investigation, Methodology, Writing—original draft.

